# Farsi Version of Social Skills Rating System-Secondary Student Form: Cultural Adaptation, Reliability and Construct Validity

**Published:** 2014

**Authors:** Ahmad Ali Eslami, Maryam Amidi Mazaheri, Firoozeh Mostafavi, Mohamad Hadi Abbasi, Ensieh Noroozi

**Affiliations:** 1 Department of Health Education and Health Promotion, School of Health, Isfahan University of Medical Sciences, Isfahan, Iran.

**Keywords:** Iranian Adolescent, Reliability, Social Skills Rating System-Secondary Students Form, Validity

## Abstract

**Objective:** Assessment of social skills is a necessary requirement to develop and evaluate the effectiveness of cognitive and behavioral interventions. This paper reports the cultural adaptation and psychometric properties of the Farsi version of the social skills rating system-secondary students form (SSRS-SS) questionnaire (Gresham and Elliot, 1990), in a normative sample of secondary school students.

**Methods:** A two-phase design was used that phase 1 consisted of the linguistic adaptation and in phase 2, using cross-sectional sample survey data, the construct validity and reliability of the Farsi version of the SSRS-SS were examined in a sample of 724 adolescents aged from 13 to 19 years.

**Results: **Content validity index was excellent, and the floor/ceiling effects were low. After deleting five of the original SSRS-SS items, the findings gave support for the item convergent and divergent validity. Factor analysis revealed four subscales. Results showed good internal consistency (0.89) and temporal stability (0.91) for the total scale score.

**Conclusion:** Findings demonstrated support for the use of the 27-item Farsi version in the school setting. Directions for future research regarding the applicability of the scale in other settings and populations of adolescents are discussed.

## Introduction

Adolescence is a developmental stage in which significant changes happen in all aspects of physical, cognitive, emotional, and social. These changes create a new feeling of identity in adolescents and lead them toward socialization with its entire emergence of developing a distinct identity (1). These features, along with a sense of attachment to the peer group, their vulnerability has been higher than other age groups. Many researchers believe that adolescence is a growing problem. Therefore, more recent research efforts have focused on increasing the competence and success factors in adolescents (2). Development of social skills leads to the emergence of the social competence and the protection of youth against risk factors (3). Social skills are observable behaviors that individual exhibits to perform competently on a social task (e.g., active listening, interpersonal relationships, the understanding of others’ feelings, etc.). Several tools have been developed to help identify the social problems in children and adolescents. The social skills rating system (SSRS) is one of the most practical means of measuring the dimensions of social skills and behavior problems in children and adolescents that it is applicable in both the normal and the abnormal (4). 

The SSRS consists of three forms for students, parents, and teachers. Each of forms of SSRS, in a separate analysis and based on data obtained from several samples from the society of America, showed a different factor structure. Factor analysis showed three factors similar (cooperation, assertiveness, and self-control) for each forms. The secondary level students form consisted of an additional factor, “Empathy” and is comprised of 39 items.

The SSRS has been widely used in diagnostic studies as a screening tool to identify individuals at risk (5-9). It has also been applied as a tool to measure outcomes of cognitive and behavioral interventions (10-14).

Internal consistency reliability of the SSRS subscales ranged from 0.51 to 0.91, with a mean of 0.75. In addition, 4-week test-retest reliability was r = 0.75 for the total scale, and ranges from 0.65 to 0.75. Criterion-related validity analysis (CBCL-YSR) revealed that correlation coefficients between SSRS and CBCL-YSR were from 0.30 to 0.72 (4). The convergent validity estimates showed significant relationships among the subscales of the SSRS forms (15). Several studies have examined factor structures of the SSRS forms (8, 16-18). Van der Oord and colleagues offered the two-factor first order model including empathy/assertiveness and self-control/cooperation (8). The findings of a recent study emphasized on the three-factor structure (self-control/cooperation, empathy and assertiveness). In this structure, items had substantial loadings on more than one factor (17). 

***The present study***

Although some versions of the SSRS, as an evaluation tool, were used in Iran (19), in reviewing the literature, using the version of SSRS-SS (age 13-18 years) did not found in an Iranian population. The purpose of the present study was to create a Farsi version of the SSRS-SS questionnaire to Iranian. Psychometric analyses were performed on the translated version by testing the floor and ceiling effects, the multi-trait scaling and exploratory factor analysis, internal consistency, and stability reliability in a normative sample of secondary school students.

In conclusion, our study can provide important evidence of the potential ability of the SSRS-SS to identify the primary social problems (which can inhibit proper development of social, emotional, educational teen does). Furthermore, the findings this study can be used in the design and evaluation of prevention programs of behavioral problems.

## Materials and Methods

***Participants***

The study design was a cross-sectional survey using stratified, two-stage, cluster sampling. Potential participants were secondary school students (grades 8-10) in Isfahan city. The sample size, with 10% probability of loss, 360 cases for each sex classes, and in total 724 cases were studied. Of the 724 participants, 41 adolescents were excluded because SSRS-SS data were incomplete. Finally, the 683 participants (334 girls and 349 boys) were included in the analysis.

***Measures***

The SSRS-SS (Gresham and Elliot 1990) is comprised of 39 items from the main domains of social skills (self-control, cooperation, empathy, and assertion). Item 11 (“I avoid doing things with others that may get me in trouble with adults”), for both the cooperation and self-control, are evaluated. The self-control subscale contains behaviors that typically emerge in conflict situations, such as responding appropriately to teasing, and in non-conflict situations that require taking turns and compromising. The cooperation subscale assesses behaviors such as sharing materials, and following rules. The empathy subscale includes behaviors that reflect concern and respect for others’ feelings and viewpoints. The assertion subscale includes initiating behaviors, such as introducing oneself and asking others for information; and a total social skills scale score is computed, with a range of 0-80. The SSRS utilizes a three point rating scale to rate the perceived frequency of social behaviors ranging from 0 to 2: 0 = “never occurs,” 1 = “sometimes occurs” and 2 = “occurs very often”.

The SSRS was standardized on a sample of 4170 children (ranged from 3^rd^- to 12^th^-graders) stratified on age, gender, geographic region, and disability status. The SSRS-SS form takes approximately 5-10 min to complete. The total scale internal consistency (0.85-0.91) and test-retest reliability (0.87) were excellent (4). In a recent study, internal consistency of the total scale equal to 0.81 reported (17).

***Procedure***

Participation of students in this study was voluntary. Students completed the self-administered questionnaire in the classroom without the teacher present (15-20 min). At the end of 4 weeks, 154 participants were asked to complete the SSRS-SS to assess the test-retest reliability.

***Data analysis***

At the first step, we translated and adapted the SSRS-SS to the Farsi language according to established guidelines (20, 21). Translation/back-translation process performed. The back translated, and original items reviewed by the research group and found to be highly similar in meaning. The content validity index (CVI) determined by an expert panel of seven members. The experts rated each item of the Farsi version of SSRS based on relevance on a Likert type ordinal scale. Finally, we pilot tested the in a classroom setting with 52 secondary school students to assess the difficulty and degree of comprehension of the questionnaire.

The next step was the psychometric validation. We tested the translated version on the 683 secondary school students. Floor and ceiling effects were estimated for global and subscales of SSRS by calculating percentages of participants that had minimum and maximum possible scores, respectively. The threshold of 20% used to define a floor or a ceiling effect. Construct validity determined by the multi-trait scaling and exploratory factor analysis. Item convergent validity examined by calculating the corrected for overlap (the recommended value above 0.4), and item divergent validity which is successful when the correlation between scores of each item and its hypothesized subscale is significantly higher (p < 0.05) than with other subscales. Results of item divergent are expressed as a percentage of scaling success.

The data examined using both a varimax and a promax rotation. The factor structure assessed using several criteria, including (a) analysis of the Eigen values greater than 1 in the Scree plot, (b) item-scale correlations ≥0.40, and (c) and cross-loadings <0.40 (22). Stability assessed in 154 adolescents using the intra-class correlation coefficient (ICC) and an ICC value of at least 0.70. Of the 154 participants, 123 completed both the test and re-test questionnaires.

## Results

***Descriptive statistics***

The sample (349 males and 334 females) consisted of students from grades 8 (53% females), 9 (47% females) and 10 (49% females). Participants’ ages ranged from 14 to 17 years (M = 15.4, SD = 0.93). The sample mean score of SSRS-SS (40 items) was 75.4 (SD = 15.61). As expected, the female group reported more scores than the male, but two means were not significantly different (t(474) = 1.12, p > 0.05). There were no also significant differences in SSRS-SS scores among grades 8-10 students (Table 1).

**Table 1:** Description of sample and comparison of mean total social skills rating system-secondary students form scale scores by grade and gender (n = 683)

	**N (%)**	**Mean ± SD**	
**8** ^th^ ** Grade**	194 (28.5)	38.70 ± 12.94	F =0.391, NS
**9** ^th^ ** Grade**	245 (36.0)	40.03 ± 12.11	
**10** ^th^ ** Grade**	242 (35.5)	40.11 ± 13.62	
**Gender**			
** Female**	334 (49)	40.47 ± 12.91	t = 1.12, NS
** Male**	349 (51)	38.94 ± 12.64	
**Total**	683 (100)	39.70 ± 12.78	

***Content validity***

The expert panel indicated that Farsi version of SSRS-SS has acceptable content validity. The means CVI (item relevance) for the final version of scale and subscales of SSRS-SS were 0.9 and higher that indicating adequate content validity (23). Of the 39 items in the tool, only item 33 from the assertion (“I start conversations with opposite-sex friends without feeling uneasy or nervous.”), omitted because of cultural problems. The review of the pilot test findings led to minor modifications in the wording of several items. Result of cultural adaptation process indicated the conceptual similarity of Persian version with the original version, and so 38 items were entered into PCA.

***Construct validity***

*Floor and ceiling effects*

Mean scores for participants’ SSRS subscales ranged between 8.8 and 10.2, and SDs, from 3.6 to 4.6. In general, floor effects were negligible (range: 1.7-4.2), and ceiling effects were low (range: 2.5-9.3) on global and four subscales of SSRS (Table 2).

*Item convergent/divergent validity*

The corrected item total correlation (CITC) for SSRS subscales ranged from 0.19 to 0.67 With regard to acceptable α values greater than 0.4 for item convergent validity, four items (assertion items 16 and 38, cooperation item 13, self-control item 19) were omitted from the tool. Although there were three higher item correlations (empathy item 39 on assertion, cooperation item 35 and assertion item 30 on self-control) with unexpected subscales, results for item divergent validity on the remaining items were relatively good, and showed that item correlations with own respective subscale were significantly greater than correlations with other subscales (Table 2). Item 11 (that assigned to two subscales in original SSRS) more closely correlated with the cooperation subscale.

***Exploratory factor analysis***

χ^2^ = 3,537.1, p < 0.001). Values of these two indicators, as well as suggest the suitability of sample size and correlation of questions for factor analysis. In the principal component analysis (PCA), the factor structure of 34 selected items with both vari- and pro-max rotations were almost the same. Due to the high correlation between factors, factor Loadings reported based on the oblique (promax) rotation (Table 3). Initial PCA with promax rotation (k = 4) yielded seven components with Eigen-values >1, accounting for 54.8% of the total variance. Three-, four-, and five-factor structures were considered as the initial solutions. The scree plot showed an elbow after factor 4 as observed a clear drop in the percent of variance (8.57-3.07%) between the fourth and fifth components. Factor 5 had only three items that its two items loaded on other factors. Thus, 4-factor solution best describes the data.

Based on the exclusion criteria, items 4, 7, 25, 27, 30, 31, and 36 were omitted from the SSRS-S-Scale because items 7, 25, 30, 31, and 36 had multiple loadings greater than 0.40, and items 4 and 27 were unrelated to any of the factors. When factor analysis was run after excluding these items using previously described parameters, four strong factors emerged that explained 47.7% of the total variance. Items had high factor loadings (>0.54), and Eigen-values were 7.01, 2.50, 2.03 and 1.23. All 27 items except item 1 and 8 were loaded on the same respective factors as in the original version. Item 1 of assertion subscale (“I make friends easily”) loaded on self-control, and item 8 of empathy subscale (“I ask friends for help with my problems”) loaded on assertion (Table 3).

**Table 2:** Structure of subscales, distributional characteristics, internal validity, and reliability of social skills rating system-secondary students form (n = 663)

**Subscale**	**No. of items (range)**	**Mean (SD)**	**Median**	**Floor (%)**	**Ceiling (%)**	**Item convergent (CITC** † **), range (S/T** ‡ **)**	**Item divergent, range (% SS** § **)**	**Alpha**
**Empathy**	10 (0-20)	9.8 (4.6)	8.0	4.2	9.3	0.40-0.65 (10/10)	0.11-0.60 (0.972)	0.87
**Self-control**	9 (0-18)	8.8 (4.0)	8.0	2.3	2.5	0.38-0.67 (8/9)	0.15-0.51 (0.972)	0.85
**Assertion**	9 (0-18)	9.6 (3.6)	10.0	1.7	4.2	0.19-0.58 (7/9)	0.05-0.72 (0.916)	0.73
**Cooperation**	10 (0-18)	10.2 (4.1)	11.0	2.3	4.5	0.26-0.62 (9/10)	0.08-0.63 (0.950)	0.83

† Corrected item total correlation;

‡ Success/total: Number of correlations exceeding the 0.40 standard/total number of correlations.

§ Scaling success: percentage of items correlating higher with their hypothesized subscale than with other SSRS subscales

The first subscale, self-control, contained eight items and accounted for 26.25% of the item variance (items 15, 22, 18, 10, 32, 34, item 1 from assertion subscale and item 35 from cooperation subscale). The second subscale, empathy, contained seven items and accounted for 9.27% of the item variance (items 29, 12, 21, 24, 28, 2, and 5). The third subscale, Assertion, contained 6 items and accounted for 7.52% of the item variance (items 3, 26, 20, 23, and items 8 and 39 from empathy subscale). The fourth subscale, cooperation, contained six items and accounted for 4.57% of the item variance (items 37, 9, 14, 17, 6, and 11).

***Reliability***

The total scale and subscales appeared to be stable and internally consistent. Internal consistency reliability of the total SSRS-SS scale was 0.89. Cronbach’s alpha coefficients for the four subscales ranged from 0.72 to 0.83 (p < 0.001). The ICC value with a 4-week interval (n = 123) was 0.88 for the total scale and varied between 0.69 and 0.81 for the four subscales (Table 4).

**Table 3:** Factor loadings and corrected item-subscale correlation for social skills rating system-secondary students form (N = 683)

**Factors and items**	**Item content**	**Loadings**	**CITC** †
**Factor 1: Self-control (8 items and score range: 0-16)**			
**15**	I do nice things for my parents like helping with household chores without being asked	0.729	0.613
**1**	I make friends easily (assertion)	0.725	0.620
**35**	I follow the teacher’s directions (cooperation)	0.695	0.589
**22**	I end fights with my parents calmly	0.688	0.582
**18**	I compromise with parents or teachers when we have disagreements	0.678	0.575
**10**	I disagree with adults without fighting or arguing	0.657	
**32**	I control my temper when people are angry with me	0.646	0.538
**34**	I take criticism from my parents without getting angry	0.631	0.521
**Factor 2: Empaty (7 items and score range: 0-14)**			
**29**	I stand up for my friends when they have been unfairly criticized	0.734	0.604
**12**	I feel sorry for others when bad things happen to them	0.712	0.592
**21**	I listen to my friends when they talk about problems they are having	0.666	0.524
**24**	I tell other people when they have done something well	0.643	0.573
**28**	I let friends know I like them by telling or showing them	0.643	0.571
**2**	I say nice things to others when they have done something well	0.615	0.494
**5**	I try to understand how my friends feel when they are angry, upset, or sad	0.535	0.404
**Factor 3: Assretion (6 items and score range: 0-12)**			
**3**	I start talks with classroom members	0.752	0.623
**26**	I start conversations with opposite-sex friends without feeling uneasy or nervous	0.744	0.618
**8**	I ask friends for help with my problems (empathy)	0.732	0.594
**20**	I ask someone I like for a date	0.727	0.673
**23**	I give compliments to members of the opposite sex	0.687	0.583
**39**	I talk things over with classmates when there is a problem or an argument.(empathy)	0.680	0.593
**Factor 4: Cooperatin (6 items and score range: 0-12)**			
**37**	I ask friends to do favors for me	0.709	0.481
**9**	I ask before using other people’s things	0.664	0.512
**14**	I keep my desk clean and neat	0.651	0.523
**17**	I finish classroom work on time	0.642	0.523
**6**	I listen to adults when they are talking with me	0.584	0.483
**11**	I avoid doing things with others that may get me in trouble with adults	0.553	0.458

† Corrected item-subscale correlation

**Table 4:** Internal consistency (n = 683), means (SD), and test-retest reliability (n = 123, after 4 weeks) of the social skills rating system-secondary students form

**Subscale (no. of items)**	**Internal consistency**	**Test, mean ± SD**	**Retest, mean ± SD**	**ICC** † ** (range)**	**P-value (95% CI** ‡ **)**
**Self-control (8) **	0.83	8.2 ± 3.6	8.3 ± 3.9	0.74 (0.64-0.81)	p < 0.0001
**Empaty (7) **	0.78	6.7 ± 3.2	7.0 ± 3.1	0.81 (0.73-0.85)	p < 0.0001
**Assretion (6) **	0.82	6.8 ± 2.8	6.9 ± 2.7	0.69 (0.58-0.77)	p < 0.0001
**Cooperatin (6) **	0.72	5.5 ± 2.3	5.6 ± 2.3	0.77 (0.68-0.83)	p < 0.0001
**Total scale (27) **	0.89	27.4 ± 8.9	27.8 ± 9.1	0.88 (0.83-0.91)	p < 0.0001

† Intra-class correlation coefficient (ICC);

‡ 95% confidence interval

## Discussion

Development of social competence and skills in children is a crucial component for positive outcomes in schools and other settings. Screening young children for developmental problems is a necessary to reduce the secondary internalizing and externalizing behavior problems. Many tools in the assessment of adolescent social competence have been developed, but few have been validated for use in an Iranian population. SSRS is developed from empirical studies in the West; it may be cultural biased when applying in the Iran situation. Cultural bias suggests that the construct under consideration has different content across different cultural groups or that individuals from different groups attach a different meaning to the construct (24). The present study provided the first empirical evaluation of the content validity, construct validity and reliability of the 27-item Farsi version the SSRS-SS in a nonclinical sample of Iranian adolescent students. The present study showed that girls scored higher than boys on social skills, but two means were not significantly different. In comparison, the several studies found that girls had significantly higher social skills scores (4, 19, 25). The Farsi version of the SSRS indicated good content validity because the CVI for the scale and subscales were 0.9 and higher. Construct validity supported in the factor analysis. The results of initial exploratory factor analysis showed that some items had low loadings (below 0.40) on their respective subscales, and cross loading above 0.40. Nevertheless, these results are in line with the previous studies. 

The present study using accurate validation procedures and removal poor items at each stage of the psychometric analysis and assigning a unexpected subscale for some items based on inspection of their loadings, a 4-factor structure with 27 items for the Persian version of the SSRS-Student form has proposed (“self-control”, “empathy”, “assertion”, and “cooperation”) that were the same as those reported by Gresham and Elliot (4). In comparison to findings of this study, Whiteside and colleague’s study was unable to identify a specific factor structure for the SSRS (18). Also, the number of previous studies reported different factor structures of the present study and the original SSRS versions (8, 17, 26). For example, the study of Van der Oord et al. (using two criteria for retention of items; factor loading of 0.35 or above, and no dual factor loading with a difference of <0.15 between the loadings on both factors) suggested a two factor structure including assertion/empathy and cooperation/self-control with 26 items for SSRS-student in a normal sample. Although in our study did not identify such two factor structure, but this study found evidence of considerable intercorrelations among items (especially among items of assertion and empathy subscales), and the existence of some substantial loadings on unexpected factors (Table 3). These results are almost identical with what reported by Mota and colleagues (2011). These researchers demonstrated the three-factor solution (self-control/cooperation, empathy, and assertion) that some of the cooperation items had strong loading on unexpected dimensions such as empathy and self-control. Four-factor structure of the present study with 27 items was explained 47.7% of the variance that is acceptable in comparison with some other studies (8, 17). Based on the results of this study, 27 items all had factor loadings higher than 0.5 and were statistically significant with a range from 0.54 to 0.75, which is similar to previous results (17, 27). Reliability analyses showed acceptable internal consistency and stability for the for the Farsi version SSRS-SS scale. Several studies have found favorable support for the SSRS in terms of internal consistency, test-retest estimates (4, 28). 

The Cronbach’s alpha value was 0.89 for the total scale and ranged from 0.72 to 0.83 for the four subscales. The corrected item-subscale correlation coefficients ranged from 0.40 (empathy item 5) to 0.67 (assertion item 20). Consistent with these findings, Mota et al. have reported internal consistency reliability of 0.87 (ranging from 0.58 to 0.72) for the total scale in a sample of 573 secondary school adolescents (17). In another study, it was equal to 0.84 and with a range from 0.51 to 0.66 for the subscales (8). In addition, ICC value between test and retest scores showed good temporal stability that suggested that the total SSRS-SS scale and subscales are precisely repeatable. Although the values of reliability and stability of the scale and subscales of SSRS-SS are high among cross-culture adaptations and validity studies, these observed results may be Due to differences in the number of factors and items. Therefore, it is difficult to compare these results with results of previous studies.

***Limitations and Future Directions***

In summary, the present study provides preliminary evidence of good psychometric properties of the 27-item Farsi version of the SSRS-SS. Although the Farsi version is relatively short and its implementation is requires less time, indeed there are important differences in the factor structure between the Farsi and the original 40-item version. Therefore, continued research is needed to develop a valid and reliable research tool and its appropriateness for the purpose of assessing social skills in different adolescent’s populations in Iran. A study using confirmatory factor analysis is needed to assess the validity of this scale in a large sample of respondents. Results showed that girls scored higher than boys on social skills. Future research should also examine the features of gender differences in adolescents’ social skills. 

## Footenote

**Declaration of interest: **None.
